# Infiltrating CCR2^+^ monocytes and their progenies, fibrocytes, contribute to colon fibrosis by inhibiting collagen degradation through the production of TIMP-1

**DOI:** 10.1038/s41598-019-45012-6

**Published:** 2019-06-12

**Authors:** Naoki Kuroda, Masahiro Masuya, Isao Tawara, Junya Tsuboi, Misao Yoneda, Kenichiro Nishikawa, Yuki Kageyama, Kensuke Hachiya, Kohshi Ohishi, Hiroshi Miwa, Reiko Yamada, Yasuhiko Hamada, Kyosuke Tanaka, Takuma Kato, Yoshiyuki Takei, Naoyuki Katayama

**Affiliations:** 1 Department of Hematology and Oncology, Mie University Graduate School of Medicine, Tsu, Mie 514-8507 Japan; 2 Department of Clinical Nutrition Medical Technology Course, Suzuka University of Medical Science, Suzuka, Mie 510-0293 Japan; 3 Department of Internal Medicine, Matsusaka City Hospital, Matsusaka, Mie 515-8544 Japan; 4 Department of Transfusion Medicine and Cell Therapy, Mie University Hospital, Tsu, Mie 514-8507 Japan; 5 Department of Gastroenterology and Hepatology, Mie University Graduate School of Medicine, Tsu, Mie 514-8507 Japan; 6 Department of Cellular and Molecular Immunology, Mie University Graduate School of Medicine, Tsu, Mie 514-8507 Japan

**Keywords:** Gastrointestinal models, Mechanisms of disease

## Abstract

Intestinal fibrosis is a serious complication in inflammatory bowel disease (IBD). Despite the remarkable success of recent anti-inflammatory therapies for IBD, incidence of intestinal fibrosis and need for bowel resection have not significantly changed. To clarify the contribution of haematopoietic-derived cells in intestinal fibrosis, we prepared bone marrow (BM) chimeric mice (chimeras), which were reconstituted with BM cells derived from enhanced green fluorescent protein (EGFP)-transgenic mice or CC chemokine receptor 2 (CCR2)-deficient mice. After 2 months of transplantation, BM chimeras were treated with azoxymethane/dextran sodium sulphate. During chronic inflammation, CCR2^+^ BM-derived monocyte and fibrocyte infiltration into the colon and CC chemokine ligand 2 production increased, leading to colon fibrosis in EGFP BM chimeras. In CCR2-deficient BM chimeras, monocyte and fibrocyte numbers in the colonic lamina propria significantly decreased, and colon fibrosis was attenuated. In colon tissue, mRNA expression of tissue inhibitor of metalloproteinase (TIMP)-1 but not of collagen I, transforming growth factor-β1 or matrix metalloproteinases was significantly different between the two chimeras. CCR2^+^ monocytes and fibrocytes showed high *Timp1* mRNA expression. Our results suggest that infiltrating CCR2^+^ monocytes and their progenies, fibrocytes, promote colon fibrosis by inhibiting collagen degradation through TIMP-1 production.

## Introduction

The incidence of inflammatory bowel disease (IBD), including ulcerative colitis and Crohn’s disease, is increasing annually. The introduction of biological agents, such as anti-tumour necrosis factor-α (TNF-α) antibody and anti-integrin antibody, provides a substantial improvement in the induction and maintaining remission of IBD. However, the progression of fibrosis in the intestinal tract and stenosis are still serious problems, and many patients require surgeries, including bowel resection and strictureplasty, for stenosis^1,2^. Therefore, the development of novel therapeutic approaches for IBD is crucial, and it is essential to examine the cellular and molecular factors that contribute to intestinal fibrosis.

Fibrosis is characterised by extensive deposition of extracellular matrix (ECM), mainly collagen I (Col I) and is caused by recurrent epithelial injuries that induce the accumulation and activation of mesenchymal cells, such as fibroblasts and myofibroblasts, as well as infiltration of inflammatory cells, such as macrophages^3–6^.

Fibroblasts are widely distributed in the interstitial space of all organs and play a critical role in regulating the turnover of ECM under normal conditions. They produce several chemokines and cytokines, such as TNF-α, transforming growth factor-β1 (TGF-β1) and interleukin-1β in response to tissue injury^4–6^. These factors recruit macrophages to the inflamed sites and activate fibroblasts to differentiate into myofibroblasts, which produce and secrete large amounts of ECM components^3–8^. Although fibroblasts and myofibroblasts are the master producers of fibrosis, it is becoming increasingly evident that monocyte-derived cells, such as fibrocytes and macrophages, also play critical roles in the pathogenesis of fibrosis^4,6,9–12^.

Fibrocytes are derived from a subset of monocytes and express mesenchymal markers, such as Col I, and haematopoietic markers, such as CD45 and CD11b^13^. They also express several chemokine receptors, such as CC chemokine receptor (CCR)1, CCR2, CCR5, CCR7, CXC chemokine receptor 4 (CXCR4) and CX3 chemokine receptor 1 (CX3CR1)^12,14–16^. They circulate in the peripheral blood (PB) and can be isolated from many fibrotic tissues^13–21^. Fibrocytes participate in both physiological wound healing and pathological fibrosis, including hypertrophic scarring, systemic sclerosis, liver cirrhosis, idiopathic pulmonary fibrosis, Grave’s eye disease, renal fibrosis and myelofibrosis^12,15–18,22–25^. In murine and human studies, fibrocytes have been reported to be associated with colon fibrosis^26,27^. It has been described that the migration of fibrocytes to injured sites involves the CC chemokine ligand 2 (CCL2)/CCR2 axis in the liver and kidney and CXC chemokine ligand 12 (CXCL12)/CXCR4 axis in the lung^16,22,24,28^. However, there are few reports concerning the role of fibrocytes and their expression of chemokine receptors related to the induction of colon fibrosis.

Macrophages produce profibrotic mediators, such as TGF-β1 and platelet-derived growth factor and activate fibroblasts into myofibroblasts^3,9^. Moreover, inflammatory macrophages in the injured sites have been demonstrated to upregulate fibroblast and myofibroblast markers, such as Col I and α-smooth muscle actin (α-SMA). Additionally, they transdifferentiate into myofibroblasts and smooth muscle-like cells during the repair process^29,30^. Macrophages also produce matrix metalloproteinases (MMPs) and tissue inhibitors of MMPs (TIMPs) as well as regulate ECM turnover^31,32^.

MMPs are a family of zinc- and calcium-dependent endopeptidases that cleave ECM components and can be sub-classified according to their substrate specificities: collagenases (MMP-1, -8 and -13), gelatinases (MMP-2 and -9), stromelysins (MMP-3 and -10) and membrane type MMPs^33–37^. MMPs are triggered by various activators, including other MMPs and proteinases, and are inactivated by specific endogenous inhibitors, such as TIMPs.

TIMPs are pleiotropic extracellular proteins. They are recognised as regulators of MMPs and can affect cell growth and differentiation, cell migration and angiogenesis independent of MMPs^33,35,38–40^. Although there are four homologous members of the TIMP family, TIMP-1 is a potent inhibitor of many MMPs. An increased level of TIMP-1 has been shown in fibrotic lesions in Crohn’s disease and a murine colitis model^7,41–43^. An imbalance due to reduced MMP activity or increased TIMP activity leads to excessive accumulation of ECM with subsequent fibrosis. TIMP-1 may be considered a target for tissue remodelling and fibrosis. Because the main cellular sources of TIMP-1 are macrophages, fibrocytes and fibroblasts, the regulation of these cells is crucial for the prevention of colon fibrosis in IBD patients^20,31,44,45^.

Recent studies have shown that CCR2 expression is essential to the recruitment of bone marrow (BM)-derived Ly6C^high^ monocytes into the inflamed colon during settings of acute colitis and that the ablation of these cells ameliorates acute inflammation^46–48^. However, it remains to be fully elucidated how CCR2^+^ monocyte-derived cells contribute to the development of colon fibrosis. To unravel this problem, we prepared BM chimeric mice (chimeras) generated from enhanced green fluorescent protein (EGFP)-transgenic mice (EGFP mice) or CCR2-deficient (CCR2^RFP/RFP^) mice and analysed which subset of CCR2^+^ monocyte-derived cells was capable of acquiring fibrotic characteristics during the development of colon fibrosis in an azoxymethane (AOM)/dextran sodium sulphate (DSS) colitis model.

## Results

### Repeated administration of DSS leads to chronic inflammation and fibrosis in the colon

**Figure 1:**
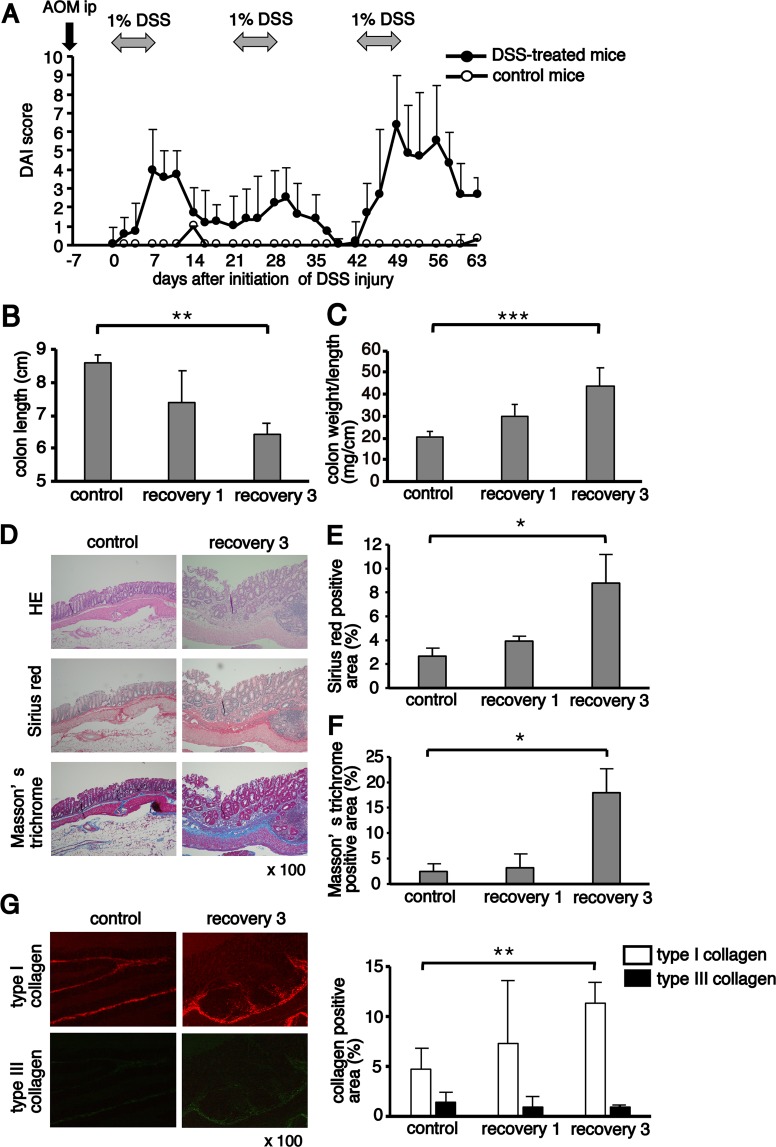
Colon fibrosis after chronic azoxymethane/dextran sodium sulphate (AOM/DSS) treatment. (**A**) Enhanced green fluorescent protein bone marrow (EGFP BM) chimeras were treated with a single intraperitoneal injection of AOM, followed by three cycles of 1% DSS for seven days in drinking water. Control mice received regular drinking water. The disease activity index (DAI) scores in DSS-treated (n = 11) and control mice (n = 3) were monitored three times per week. (**B**,**C**) The colon length and weight to length ratio were measured on days 17 (recovery 1) and 59 (recovery 3) from the start of DSS. The sample size for each group was n = 6. (**D**) Paraffin-embedded colon sections obtained from untreated (control) mice (n = 3) and chronically DSS-treated (recovery 3) mice (n = 3) were stained with haematoxylin and eosin (HE; upper), Sirius red (middle) and Masson’s trichrome (lower). Original magnification, ×100. (**E**,**F**) Quantification of Sirius red- and Masson’s trichrome-positive areas. (**G**) Sections stained with Sirius red were analysed using polarised microscopy to quantify type I and type III collagen. Quantification of type I and type III collagen-positive areas. The histograms show the mean percentage of the staining area per total colon area. The experiments were performed at least twice, yielding similar results. Data are expressed as the mean ± SD. ^*^p < 0.05, ^**^p < 0.01 and ^***^p < 0.001 by Kruskal–Wallis test followed by Dunn’s multiple comparison test.

To facilitate the observation of BM-derived cells infiltrating the injured colon, we generated EGFP BM chimeras. At 2 months after BM transplantation, the BM chimeras were treated with AOM/DSS. Chronic colitis was induced by repeated administration of 1% DSS. Disease activity index (DAI) increased after the administration of 1% DSS, and the score was higher in the third cycle than in the first cycle (Fig. 1A). Treated BM chimeras were sacrificed on day 17 (recovery 1) from the start of DSS administration and on day 59 (recovery 3). The colon length was significantly shorter, and the colon weight to length ratio was significantly higher in mice after the third cycle of DSS compared with the untreated control BM chimeras (Fig. 1B,C).

Histological analysis of colon sections with haematoxylin and eosin (HE) stains revealed areas with severe mucosal and submucosal inflammatory cell infiltration and mucosal thickening but not dysplasia or cancer on day 59 (recovery 3) (Fig. 1D). Colon sections were stained with Sirius red and Masson’s trichrome to investigate the colon fibrosis in the chronic DSS-treated mice. Collagen deposition, which was shown as a percentage of Sirius red-positive red areas and Masson’s trichrome-positive blue areas, significantly increased in the colon tissue of mice after the third cycle of DSS compared with the untreated control mice (Fig. 1D–F). The distribution of type I and type III collagen was determined in Sirius red-stained colon sections using a polarised light microscope, in which type I and type III collagen were observed as red and green colours, respectively (Fig. 1G). Although the percentage of type I collagen significantly increased in the colon tissue of mice after the third cycle of DSS compared with the untreated control BM chimeras, that of type III collagen did not change during the development of colitis.

### Chronic inflammation increases the number of BM-derived cells in the colon

**Figure 2:**
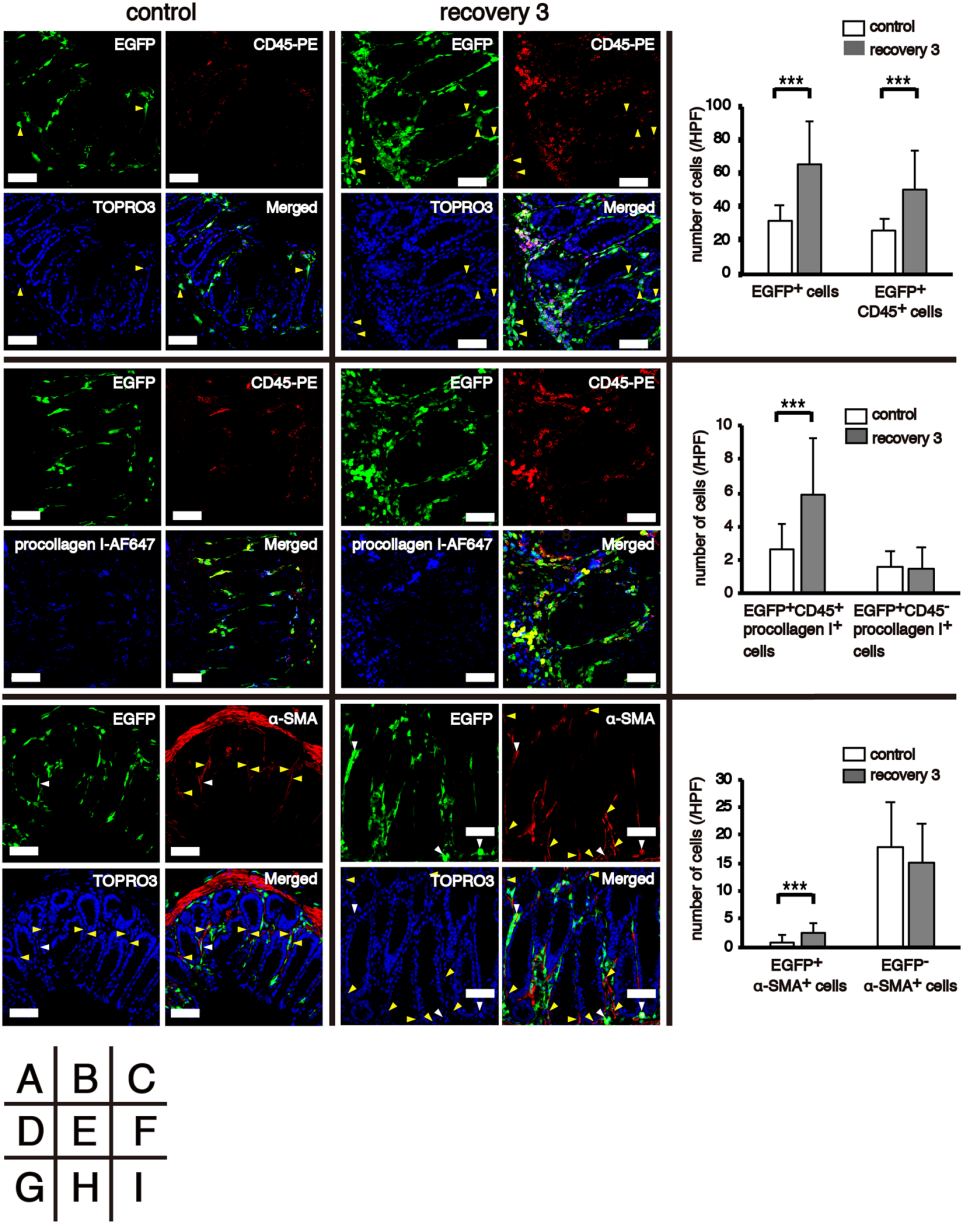
Identification of bone marrow (BM)-derived cells in the colon. Frozen colon sections obtained from untreated (control; n = 3) and chronically treated (recovery 3; n = 3) EGFP BM chimeras were subjected to immunofluorescent staining. (**A**,**B**) The panels show EGFP as green, CD45 as red and TOPRO3 as blue. Yellow triangles indicate EGFP^+^CD45^−^ cells. (**C**) The histograms show the mean number of EGFP^+^ cells and EGFP^+^CD45^+^ cells in a high-power field (HPF). (**D**,**E**) The panels show EGFP as green, CD45 as red and procollagen I as blue. (**F**) The histograms show the mean number of EGFP^+^CD45^+^procollagen I^+^ and EGFP^+^CD45^−^procollagen I^+^ cells in a HPF. (**G**,**H**) The panels show EGFP as green, α-SMA as red and TOPRO3 as blue. White and yellow triangles indicate EGFP^+^α-SMA^+^ and EGFP^−^α-SMA^+^ cells, respectively. (**I**) The histograms show the mean number of EGFP^+^α-SMA^+^ and EGFP^−^α-SMA^+^ cells in a HPF. The experiments were performed at least twice and yielded similar results. Data are expressed as the mean ± SD. ^***^p < 0.001 by Mann–Whitney U test. Original magnification, ×400. Scale bars, 50 µm.

Fluorescence immunohistochemical staining of frozen colon sections from EGFP BM chimeras was performed to investigate the infiltration of BM-derived cells into the colon. EGFP^+^ cells were mainly detected in the mucosa of the colon in untreated control mice and the mucosa and submucosa of the colon in mice after the third cycle of DSS treatment. The number of EGFP^+^ cells and EGFP^+^CD45^+^ haematopoietic cells increased two-fold after the third cycle of DSS treatment compared with the untreated control mice (Fig. 2A–C). EGFP^+^CD45^+^procollagen I^+^, EGFP^+^CD45^−^procollagen I^+^ and EGFP^+^α-SMA^+^ cells are considered BM-derived fibrocytes, BM-derived fibroblast-like cells including myofibroblasts and BM-derived myofibroblasts, respectively. Although numbers of these three fractions in the untreated control mice were low, EGFP^+^CD45^+^procollagen I^+^ fibrocytes and EGFP^+^α-SMA^+^ cells significantly increased after the third cycle of DSS (Fig. 2D–I). However, a vast majority of α-SMA^+^ myofibroblasts in the colonic lamina propria (LP) were resident cells, and there was no change in the total number (BM-derived EGFP^+^α-SMA^+^ and resident EGFP^−^α-SMA^+^ cells) between the untreated control mice and mice after the third cycle of DSS treatment (Fig. 2I).

### BM-derived monocytes and fibrocytes significantly increased in the inflamed colon

**Figure 3:**
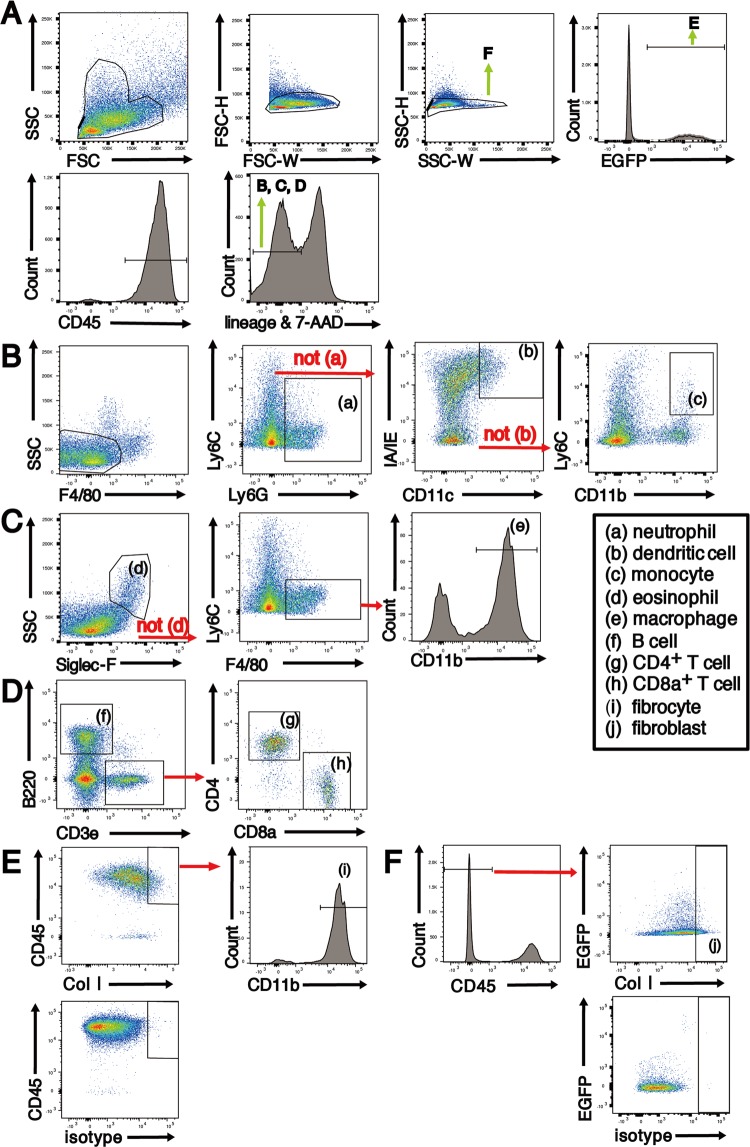
Representative flow cytometry gating strategy for the identification of major immune cell populations in the colon LP of EGFP BM chimeras. (**A**) Isolated colon LP cells were first gated on size, singularity and positive expression of EGFP and CD45. Next, lineage- and 7-AAD positive cells were eliminated. The lineage antibody cocktail was different depending on the cell type that was analysed. (**B**) The lineage antibody cocktail for cell surface staining of (a) neutrophils, (b) dendritic cells and (c) monocytes included anti-B220, anti-CD3e, anti-NK1.1, anti-TER119 and anti-Siglec F antibodies. (**C**) The lineage antibody cocktail for (d) eosinophils and (e) macrophages included anti-B220, anti-CD3e, anti-NK1.1 and anti-TER119 antibodies. (**D**) No lineage antibody cocktail was used for cell surface staining of (f) B cells, (g) CD4^+^ T cells and (h) CD8^+^ T cells. (**E**,**F**) To identify fibrocytes and fibroblasts, intracellular staining for Col I and surface staining for CD45 and CD11b were performed. BM-derived fibrocytes (i) and fibroblasts (j) were identified as EGFP^+^CD45^+^CD11b^+^Col I^+^ and CD45^−^Col I^+^ cells, respectively.

**Figure 4:**
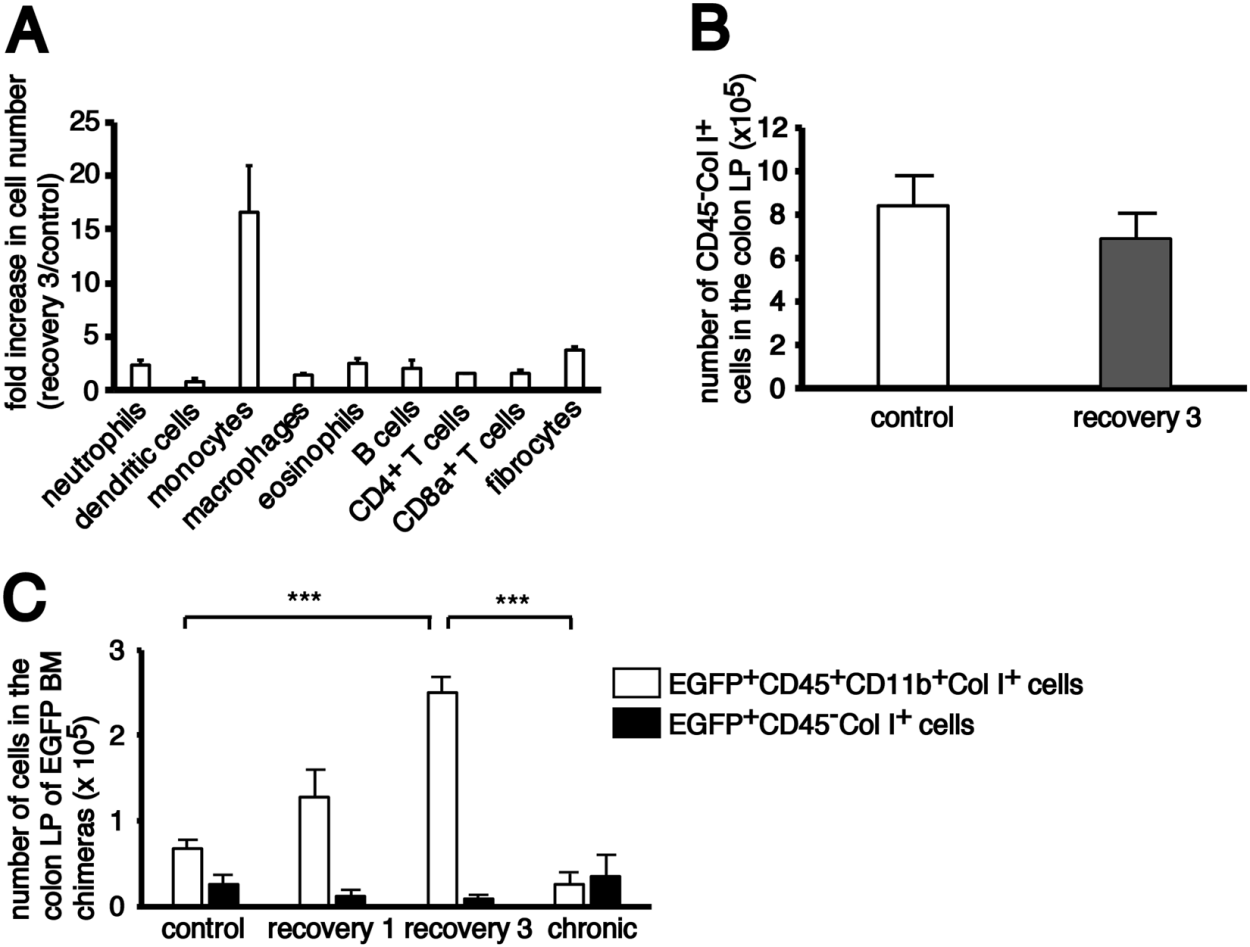
Flow cytometry analysis of BM-derived cells in the colonic lamina propria (LP). (**A**) Fold increase in cell number of different cell fractions in the colonic LP of recovery 3 mice (n = 3) compared with control mice (n = 3). The quantitative data were obtained using flow cytometry. (**B**) The histograms show the mean number of total CD45^−^collagen I^+^ cells in the colonic LP of both control and recovery 3 mice. (**C**) The histograms show the mean number of BM-derived EGFP^+^CD45^+^CD11b^+^Col I^+^ fibrocytes and EGFP^+^CD45^−^Col I^+^ fibroblasts in the colonic LP of EGFP BM chimeras chronically treated with AOM/DSS. The sample size for each group was n = 3. The experiments were performed twice and yielded similar results. Data are expressed as mean ± SD. ^***^p < 0.001 statistical significance using Kruskal–Wallis test followed by Dunn’s multiple comparison test.

Next, we examined which fraction of the BM-derived cells infiltrated the colonic LP after chronic DSS treatment using flow cytometry (Fig. 3A–F). After the third DSS treatment, CD45^+^F4/80^−^Ly6G^−^CD11c^−^CD11b^+^Ly6C^+^ monocytes and CD45^+^CD11b^+^Col I^+^ fibrocytes increased 16.6-fold and 3.8-fold, respectively, but other fractions increased by less than 2.5-fold in the colonic LP of EGFP BM chimeras (Fig. 4A). There was no difference in the number of CD45^−^Col I^+^ cells, which include fibroblasts and myofibroblasts, between the untreated control mice and mice after the third cycle of DSS treatment (Fig. 4B).

Most CD45^−^Col I^+^ cells were negative for EGFP, suggesting the origin from resident cells (Fig. 3F). On the other hand, fibrocytes detected in the colonic LP were mostly derived from BM haematopoietic cells because the frequency of EGFP^+^ cells was 95%–97% in the untreated control and chronically treated mice (data not shown). The number of EGFP^+^CD45^+^CD11b^+^Col I^+^ fibrocytes in the colonic LP of chronically treated EGFP BM chimeras significantly increased in association with the development of colon inflammation and were markedly reduced 2 months after cessation of DSS treatment (chronic phase). The number of EGFP^+^CD45^−^Col I^+^ cells was much lower than the number of EGFP^+^CD45^+^CD11b^+^Col I^+^ fibrocytes and did not change during the development of colitis (Fig. 4C).

The chemokine receptor expression of monocytes and macrophages in the colonic LP and fibrocytes in the colonic LP as well as PB and chemokine production in the colon were analysed to investigate which chemokine influences the infiltration of BM-derived monocyte-lineage cells into the colonic LP. Of note, monocyte-lineage cells express chemokine receptors, such as CCR2, CX3CR1 and CXCR4^49–51^. Therefore, the expression of CCR2 and CX3CR1 was examined using CCR2^RFP/+^CX3CR1^GFP/+^ hybrid mice, in which CCR2^+^ cells express red fluorescent protein and CX3CR1^+^ cells express green fluorescent protein^52^. Additionally, CXCR4 expression was assessed using an antibody against CXCR4 during the first cycle of DSS treatment.

**Figure 5:**
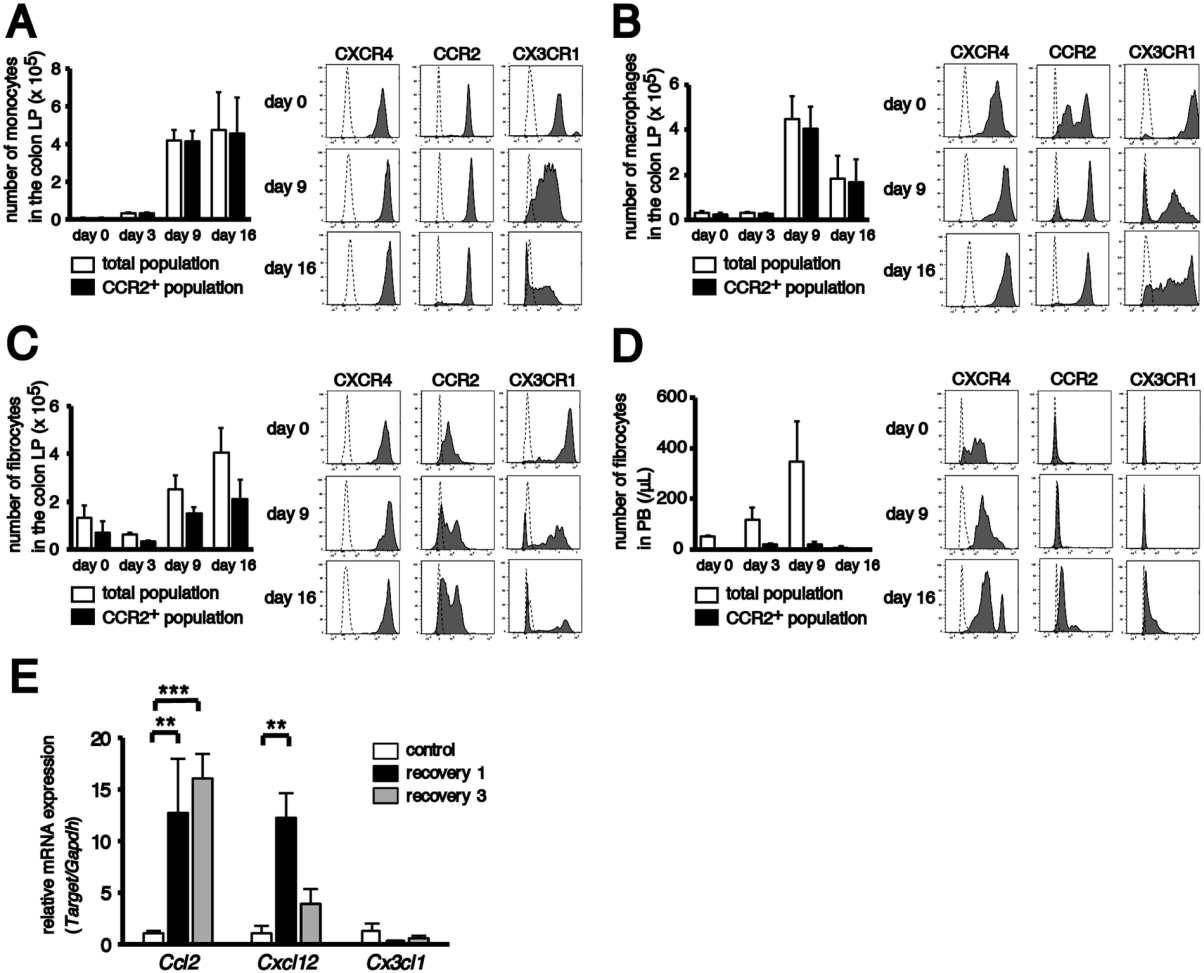
Expression of chemokine receptors on monocyte-lineage cells and chemokines in the colon. Colons and peripheral blood (PB) were harvested from non-transplanted CCR2^RFP/+^CX3CR1^GFP/+^ hybrid mice on days 0, 3, 9 and 16 after the initiation of 2% DSS treatment. The quantitative data were obtained using flow cytometry. The sample size for each group was n = 3. (**A–D**) The histograms in the left panels indicate the mean number of (**A**) monocytes, (**B**) macrophages, (**C**) fibrocytes in the colonic LP and (**D**) fibrocytes in the PB. Open bars, total population; closed bars, CCR2^+^ population. Representative flow cytometry analysis of the expression of CXCR4, CCR2 and CX3CR1 in each fraction are shown in the right panels. Dashed lines indicate data using the isotype control or background autofluorescence staining in WT mice. (**E**) The histograms show relative mRNA expression of *Ccl2*, *Cxcl12* and *Cx3cl1* in colon tissues obtained from untreated (control), acutely treated (recovery 1) and chronically treated (recovery 3) EGFP BM chimeras. The sample size for each group was n = 3. Data are expressed as the mean ± SD. ^**^p < 0.01 and ^***^p < 0.001 statistical significance using Kruskal–Wallis test followed by Dunn’s multiple comparison test. The experiments were performed three times and yielded similar results.

First, the number of each cell type was counted on days 0, 3, 9 and 16 after DSS treatment initiation. The total monocytes and fibrocytes in the colonic LP increased in a time-dependent manner (days 9–16) and peaked at day 16. Macrophages peaked on day 9 and subsequently declined to approximately half of their peak value on day 16. Fibrocytes in PB increased in a time-dependent manner (days 3–9) and were markedly reduced from day 9 to day 16 (Fig. 5A–D).

In monocytes, CXCR4, CCR2 and CX3CR1 were highly expressed before DSS treatment. Although the expression level of both CXCR4 and CCR2 did not change, CX3CR1^low/−^ cells increased with the development of colitis and approximately half of the monocytes were negative for CX3CR1 on day 16 (Fig. 5A). In comparison with monocytes, macrophages expressed a higher level of CX3CR1 and a similar level of CXCR4 before DSS treatment. CCR2 was bimodally expressed. Although the expression level of CXCR4 did not change, almost all macrophages became highly positive for CCR2 with the development of colitis. The percentage of CX3CR1^−^ cells in macrophages transiently increased on day 9 after DSS treatment initiation but decreased from days 9 to 16 (Fig. 5B). The expression pattern of CXCR4 and CX3CR1 on fibrocytes in the colonic LP was similar to that on monocytes and macrophages before DSS treatment. The expression level of CCR2 on fibrocytes was lower than that on monocytes and macrophages. The CXCR4 expression did not change, however, CX3CR1^−^ fibrocytes gradually increased with the development of colitis. The CCR2 expression became bimodal (CCR2^−^ and CCR2^+^) after DSS treatment (Fig. 5C). Fibrocytes in the PB only expressed CXCR4, but the expression level was lower than that on fibrocytes in the colonic LP. The expression of both CCR2 and CX3CR1 remained almost negative even after DSS treatment (Fig. 5D). These results indicate the possibility that fibrocytes in the colonic LP are composed of two types of cells: CCR2^+^ cells, which are phenotypically similar to monocytes and macrophages in the colonic LP, and CCR2^−^ cells, which are probably fibrocytes in the PB.

Chemokine production in colon tissue before and after DSS treatment was analysed by quantitative real-time reverse transcription polymerase chain reaction (RT-PCR). The mRNA expression of *Ccl2* significantly increased in a time-dependent manner after chronic injury (Fig. 5E). Although the expression of *Cxcl12* significantly increased after the first cycle of DSS treatment, it subsequently declined during further treatment. The expression of *Cx3cl1* was not altered during the experiment.

### Fibrocytes in the colonic LP are derived from both CCR2^+^ infiltrating monocytes and CCR2^−^ circulating fibrocytes

**Figure 6:**
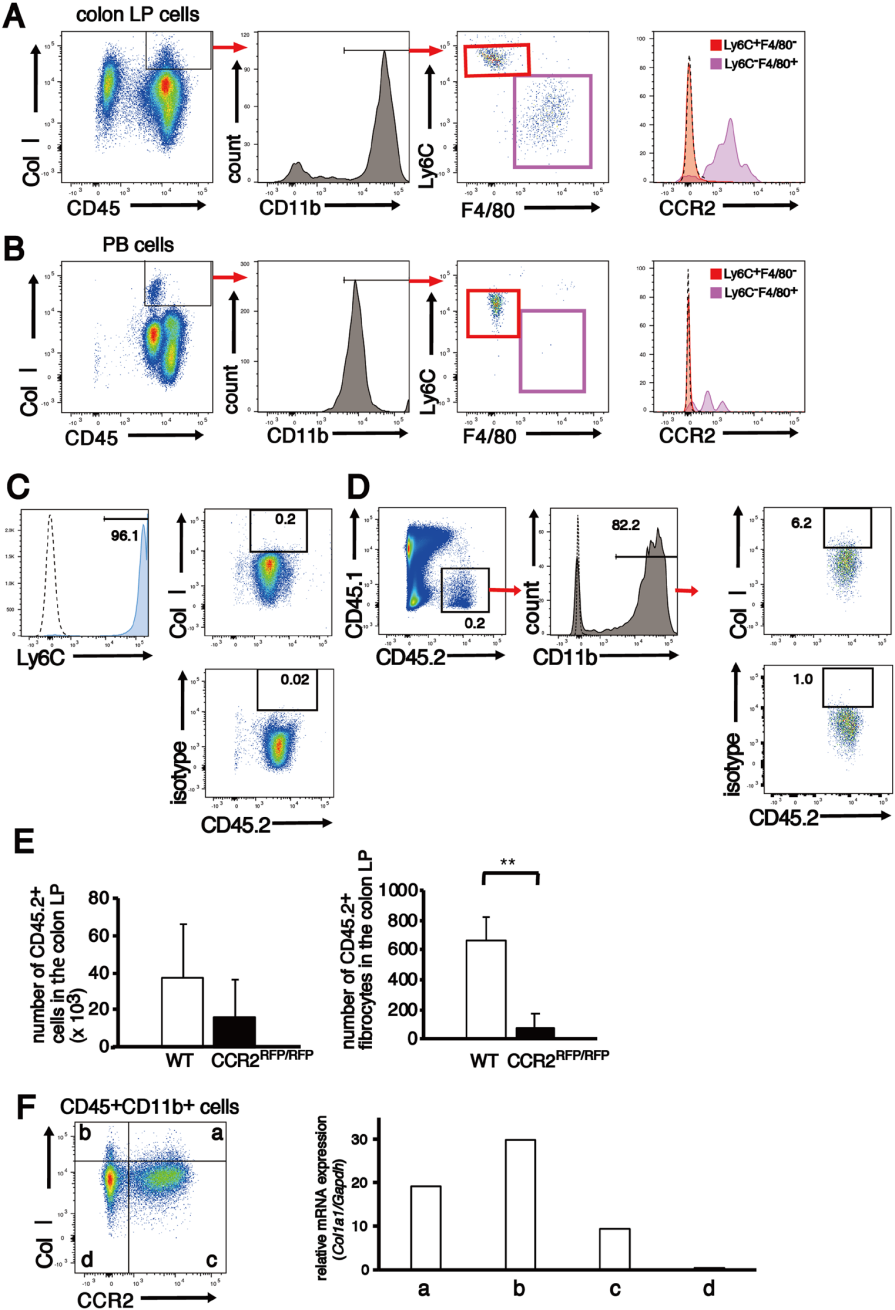
Identification of two distinct fibrocytes in the colonic LP. (**A**,**B**) Colons and PB were harvested from non-transplanted CCR2^RFP/+^CX3CR1^GFP/+^ hybrid mice (n = 6) on day16 after the initiation of 2% DSS treatment. Representative flow cytometry analysis of the expression of Ly6C, F4/80 and CCR2 on CD45^+^CD11b^+^Col I^+^ cells in the colonic LP (**A**) and PB (**B**) are shown. (**C–E**) Adoptive transfer experiments. (**C**) Ly6C^+^ monocytes isolated from BM of C57BL/6J-Ly5.2 mice were negative for Col I. (**D**) Adoptively transferred CD45.2^+^Ly6C^+^ monocytes partly differentiated into Col I^+^ fibrocytes in the injured colon. Results are representative of two independent experiments. (**E**) Ly6C^+^ monocytes isolated from BM of CCR2^RFP/RFP^ mice (C57BL/6J-Ly5.2 background) neither engrafted into the injured colon nor differentiated into fibrocytes in the colonic LP. The sample size for each group was n = 6. (**F**) CD45^+^CD11b^+^ LP cells obtained from seven pooled colons of EGFP BM chimeras chronically treated with 1% DSS were divided into four subpopulations: (a) CCR2^+^ fibrocytes, (b) CCR2^−^ fibrocytes, (c) CCR2^+^Col I^−^ monocytes/macrophages and (d) CCR2^−^Col I^−^ myeloid cells by Col I and CCR2 expression and they were sorted using FACSAria II. The right panel shows the mRNA expression level of *Col1a1* in each subpopulation. Data are normalised to *Gapdh*. The experiments were performed three times and yielded similar results. Data are presented as the mean ± SD. ^**^p < 0.01 by Mann–Whitney U test.

Fibrocytes in colonic LP were subdivided into two populations—Ly6C^high^F4/80^−^ and Ly6C^low/−^F4/80^+^ cells—using cell surface expression of Ly6C and F4/80. Ly6C^high^F4/80^−^ cells were negative for CCR2, and Ly6C^low/−^F4/80^+^ cells were positive for CCR2 (Fig. 6A). However, fibrocytes in the PB were mostly Ly6C^high^F4/80^−^CCR2^−^ (Fig. 6B). These results confirmed the results presented in Fig. 5C and D.

To clarify whether BM-derived CCR2^+^Ly6C^+^ monocytes are precursors of fibrocytes in the colonic LP, we adoptively transferred 3–4 × 10^6^ Ly6C^high^CD45.2^+^Col I^−^ monocytes, which were isolated from BM total nucleated cells (TNCs) of wild-type (WT) C57BL/6J-Ly5.2 or CCR2^RFP/RFP^ mice, into C57BL/6J-Ly5.1 mice on days 8 and 13 after DSS treatment initiation (Fig. 6C). We analysed the appearance of donor monocyte-derived CD45.2^+^CD11b^+^Col I^+^ fibrocytes in the colonic LP on day 16. Figure 6D shows that 6.2% of the CD45.2^+^CD11b^+^ cells were positive for Col I in mice that received Ly6C^high^ monocytes from WT mice. The absolute number of CD45.2^+^CD11b^+^Col I^+^ fibrocytes in the colonic LP was significantly reduced in mice that received Ly6C^high^ monocytes from CCR2^RFP/RFP^ mice, compared with those that received cells from WT mice (Fig. 6E). These results demonstrate that BM-derived Ly6C^+^CCR2^+^ monocytes migrated to the injured colon and a part of them differentiated into CCR2^+^ fibrocytes.

Previous reports have demonstrated that uptake of secreted Col I by haematopoietic cells accounted for the fibrocyte population, and the presence of intracellular Col I does not necessarily indicate Col I production by fibrocytes^53–55^. Therefore, we sorted colonic LP cells, which were isolated from pooled colons of seven non-transplanted C57BL/6J-Ly5.1 mice following three cycles of treatment with 1% DSS, into four populations of CD45^+^CD11b^+^ cells based on Col I and CCR2 staining and examined *Col1a1* expression (Fig. 6F). *Col1a1* was detected in (a) Col I^+^CCR2^+^ cells and (b) Col I^+^CCR2^−^ cells as well as (c) Col I^−^CCR2^+^ cells and the expression level was higher in Col I^+^CCR2^+^ and Col I^+^CCR2^−^ cells than in Col I^−^CCR2^+^ cells.

Col I^−^CCR2^+^ cells were mainly composed of monocytes and macrophages. These results suggest that some CCR2^+^ monocytes and macrophages in the colonic LP can produce Col I and are precursors of CCR2^+^Col I^+^ fibrocytes. Therefore, fibrocytes in the colonic LP are thought to consist of two subsets: CCR2^+^ infiltrating monocyte-derived fibrocytes and CCR2^−^ circulating fibrocytes.

### CCR2 deficiency attenuates the development of colon fibrosis

**Figure 7:**
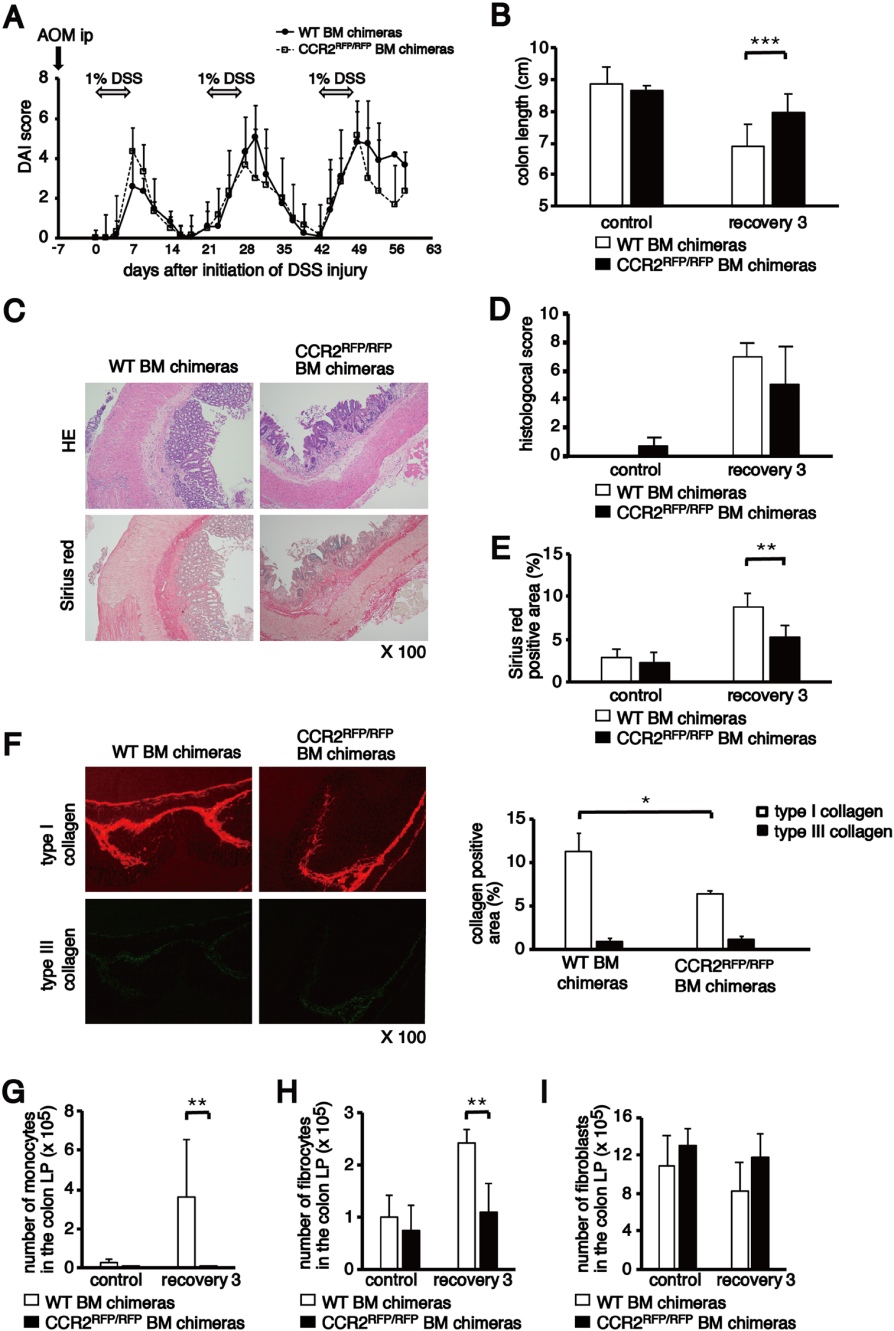
Reduction of colon fibrosis after AOM/DSS treatment by CCR2 deletion. (**A**) Change of DAI score in WT BM (n = 17) and CCR2^RFP/RFP^ BM chimeras (n = 6) after AOM/DSS treatment. (**B**) Comparison of colon length between WT BM (n = 8) and CCR2^RFP/RFP^ BM chimeras (n = 6 or 7) treated with or without 1% DSS. (**C**) Histological analysis of colon sections from WT BM and CCR2^RFP/RFP^ BM chimeras on day 59 (recovery 3). Representative pictures of colon sections stained with HE (upper) and Sirius red (lower). Original magnification, ×100. (**D**) Comparison of histological score between WT BM (n = 6) and CCR2^RFP/RFP^ BM chimeras (n = 6) treated with or without 1% DSS. (**E**) Quantification of Sirius red-positive areas in the colon of WT BM (n = 6) and CCR2^RFP/RFP^ BM chimeras (n = 6) treated with or without AOM/DSS. (**F**) Quantification of collagen-positive areas in the colon of the WT BM (n = 3) and CCR2^RFP/RFP^ BM chimeras (n = 3) treated with or without AOM/DSS. (G, H, I) The numbers of monocytes, fibrocytes and fibroblasts in the colon LP of WT BM and CCR2^RFP/RFP^ BM chimeras treated with or without AOM/DSS were analysed using flow cytometry. The sample size for each group was n = 6. The experiments were performed at least twice, yielding similar results. Data are presented as the mean ± SD. ^*^p < 0.05, ^**^p < 0.01 and ^***^p < 0.001 by Mann–Whitney U test.

Based on the above observations, it was considered that the CCL2/CCR2 axis is essential to recruit Ly6C^+^CCR2^+^ monocytes to the injured colon and induce colon fibrosis via the accumulation of CCR2^+^ fibrocytes. Therefore, we prepared WT BM and CCR2^RFP/RFP^ BM chimeras and treated them with three cycles of DSS treatment. Although a significant increase in the DAI score was observed in WT BM and CCR2^RFP/RFP^ BM chimeras, no difference was found between both mice (Fig. 7A). The colon length decreased in both BM chimeras after DSS treatment. Shortening of the colon length was dampened in the CCR2^RFP/RFP^ BM chimeras compared with that in the WT BM chimeras; however, the colon length after the third cycle of DSS treatment was shorter than that before DSS treatment in the CCR2^RFP/RFP^ BM chimeras (Fig. 7B). The histological inflammation score increased after chronic DSS treatment in the WT BM and CCR2^RFP/RFP^ BM chimeras and was not significantly different between both BM chimeras (Fig. 7C,D).

Colon fibrosis was assessed by Sirius red staining. Chronic DSS treatment led to a mild colonic inflammation and submucosal oedema with extensive deposition of collagen in WT BM chimeras (Fig. 7C). In contrast, CCR2^RFP/RFP^ BM chimeras showed a significant reduction of colon fibrosis (Fig. 7C,E). The percentage of type I collagen but not that of type III collagen significantly decreased in the CCR2^RFP/RFP^ BM chimeras compared with the WT BM chimeras after the third DSS treatment (Fig. 7F).

After chronic DSS administration, the number of monocytes in the colonic LP markedly increased in WT BM chimeras compared with untreated control conditions. However, there were almost no monocytes in both untreated and chronically treated CCR2^RFP/RFP^ BM chimeras (Fig. 7G). Although a marked increase of fibrocytes in the colonic LP was seen in chronically treated WT BM chimeras compared with corresponding control mice, no difference was observed in the number of fibrocytes between untreated and chronically treated CCR2^RFP/RFP^ BM chimeras (Fig. 7H). The number of monocytes and fibrocytes in the colonic LP of chronically treated CCR2^RFP/RFP^ BM chimeras were extremely lower than those in the colonic LP of WT BM chimeras. However, there was no difference in the number of fibroblasts in the colonic LP before and after chronic DSS treatment in both BM chimeras (Fig. 7I). These results indicate that targeted deletion of CCR2 in BM-derived cells attenuated colon fibrosis by inhibiting the accumulation of monocytes and fibrocytes in the inflamed colon.

### BM-derived CCR2^+^ monocytes and fibrocytes promote colon fibrosis through the production of TIMP-1

Tissue fibrosis occurs when collagen synthesis surpasses collagen degradation, which is regulated by MMPs and TIMPs^7,34,35,40^. Our present results demonstrate that BM-derived CCR2^+^ monocytes and fibrocytes play an essential role in the development of colon fibrosis. To clarify how these cells contribute to colon fibrosis, we analysed the expression of several genes involved in collagen synthesis and collagen degradation within DSS-treated colon tissues of both EGFP BM and CCR2^RFP/RFP^ BM chimeras.

**Figure 8:**
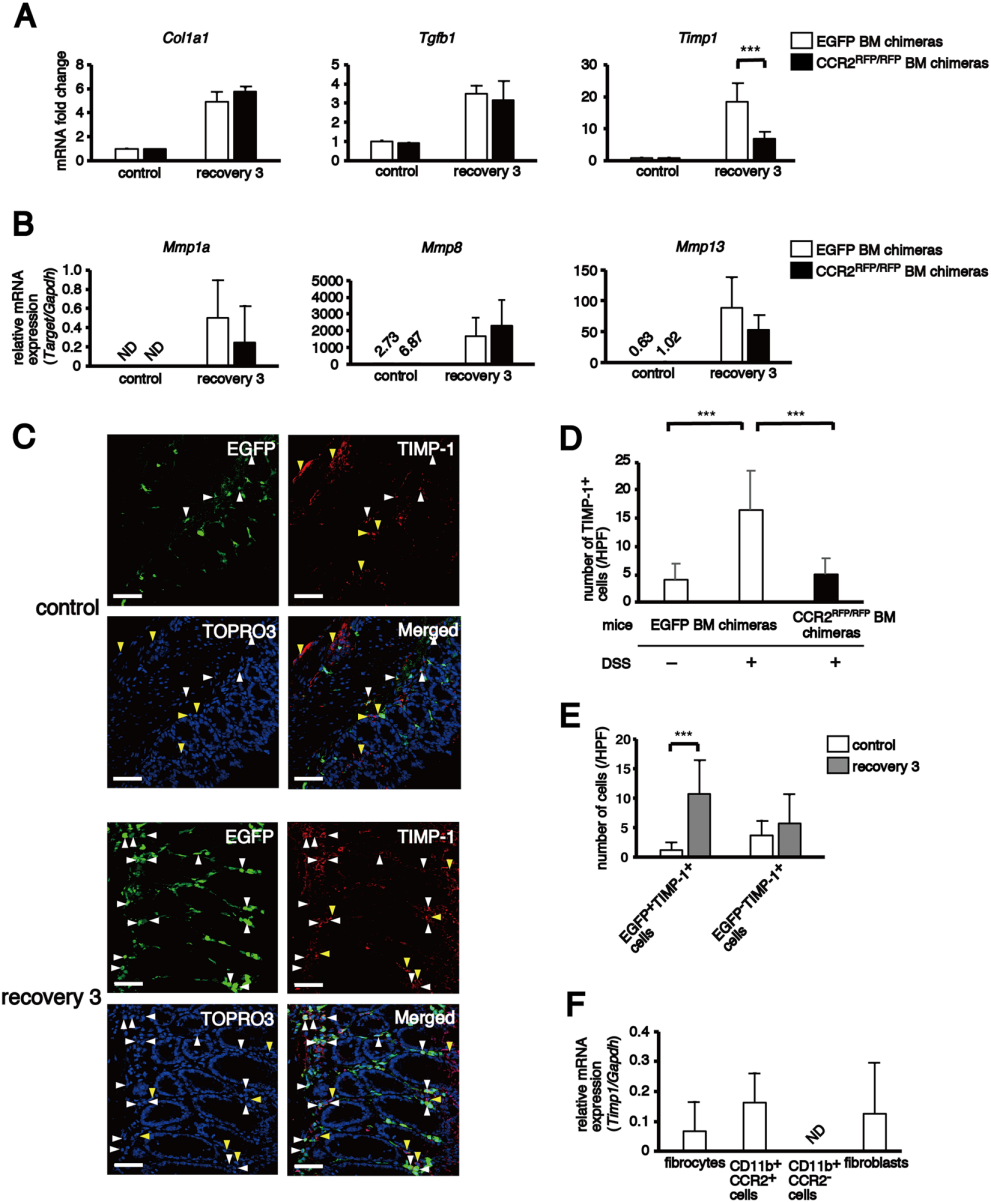
Reduction of TIMP-1 expression by CCR2 deletion. Expression levels of various genes in the colon were analysed by quantitative real-time reverse transcription polymerase chain reaction. (**A**) Expression levels of *Col1a1*, *Tgfb1* and *Timp1* relative to *Gapdh* in colon tissue were calculated and normalised to the expression levels of EGFP BM chimeras without injury. (**B**) Expression levels of *Mmp1a*, *Mmp8* and *Mmp13* in colon tissue were normalised to *Gapdh*. (**C**) The panels show EGFP as green, TIMP-1 as red and TOPRO3 as blue. White and yellow triangles indicate EGFP^+^TIMP-1^+^ and EGFP^–^TIMP-1^+^ cells, respectively. (**D**) The histograms show the mean numbers of TIMP-1^+^ cells per HPF in the colon tissues of EGFP BM and CCR2^RFP/RFP^ BM chimeras. (**E**) The histograms show the mean numbers of EGFP^+^TIMP-1^+^ and EGFP^−^TIMP-1^+^ cells per HPF in the colon tissues of EGFP BM chimeras. (**F**) The expression levels of *Timp1* in four cell populations in the colon LP. The gating strategy was as follows: fibrocytes; CD45^+^CD11b^+^Col I^+^ cells, CD11b^+^CCR2^+^ cells; CD45^+^CD11b^+^Col I^−^CCR2^+^ cells, CD11b^+^CCR2^−^ cells; CD45^+^CD11b^+^Col I^−^CCR2^-^ cells, fibroblasts; CD45^−^Col I^+^ cells. The sample size for each group was n = 6. The experiments were performed at least twice, yielding similar results. Data are expressed as the mean ± SD. ^***^p < 0.001 by Mann–Whitney U test and Kruskal–Wallis test followed by Dunn’s multiple comparison test.

First, we analysed the expression of *Col1a1*, *Tgfb1*, *Timp1*, *Mmp1a*, *Mmp8* and *Mmp13* in the rectal tissues. Although the expressions of *Col1a1* and *Tgfb1* were upregulated after chronic DSS treatment in EGFP BM and CCR2^RFP/RFP^ BM chimeras, there were no significant differences between both chimeras (Fig. 8A). A significant reduction of colon fibrosis, which was observed in CCR2^RFP/RFP^ BM chimeras, was not caused by the inhibition of TGF-β1-induced Col I synthesis in the inflamed colon.

There are three types of collagenase: interstitial collagenase (MMP-1, collagenase 1), neutrophil collagenase (MMP-8, collagenase 2) and collagenase 3 (MMP-13)^34–37^. In mice, two MMP-1 homologues, MMP-1a and MMP-1b, are identified, and MMP-1a has a collagenase activity^56^. These three collagenases have a high collagenolytic activity and are associated with collagen degradation. Although the expressions of *Mmp1a*, *Mmp8* and *Mmp13* within the colon tissues of EGFP BM and CCR2^RFP/RFP^ BM chimeras were upregulated after chronic DSS treatment, there were no significant differences between both the chimeras (Fig. 8B).

The expression of *Timp1* was upregulated after DSS treatment in both EGFP BM and CCR2^RFP/RFP^ BM chimeras, but the expression level in EGFP BM chimeras was significantly higher than in CCR2^RFP/RFP^ BM chimeras (Fig. 8A). Moreover, the number of TIMP-1^+^ cells in the colon significantly increased after chronic DSS treatment in EGFP BM chimeras (Fig. 8C,D). However, the amount in CCR2^RFP/RFP^ BM chimeras was similar to the untreated control EGFP BM chimeras (Fig. 8D). Although the number of BM-derived EGFP^+^TIMP-1^+^ cells significantly increased after chronic DSS treatment, resident EGFP^−^TIMP-1^+^ cells did not change (Fig. 8C,E).

Next, we isolated CD45^+^CD11b^+^Col I^+^ fibrocytes, CD11b^+^CCR2^+^Col I^−^ cells, CD11b^+^CCR2^−^Col I^−^ cells and CD45^−^Col I^+^ fibroblasts (including myofibroblasts) from the colonic LP cells of the EGFP BM chimeras after three cycles of DSS treatment to identify the cell populations producing TIMP-1 in the colon. The expression of *Timp1* was detected in fibrocytes, CD11b^+^CCR2^+^Col I^−^ cells and fibroblasts (Fig. 8F). These results demonstrate that TIMP-1 produced by CCR2^+^ cells, such as fibrocytes, monocytes and macrophages but not by resident fibroblasts within the colonic LP, may be involved in the development of colon fibrosis.

## Discussion

Recent murine and human studies have demonstrated that fibrocytes accumulate in the colonic submucosa and directly augment fibrosis by producing Col I^26,27^. However, the detailed functional analysis of fibrocytes has not been entirely performed. In the current study, we used BM chimeras, which were transplanted with BM-TNCs obtained from EGFP or CCR2^RFP/RFP^ mice, to investigate which cells are precursors of fibrocytes in the colon, how they migrate into the inflamed colon and how they contribute to colon fibrosis.

We identified two types of fibrocytes, CCR2^+^ and CCR2^−^ fibrocytes, in the colonic LP of mice chronically treated with DSS. Although the former cells were Ly6C^−^F4/80^+^ and this phenotype was similar to that of macrophages, the latter cells were Ly6C^high^F4/80^−^ and similar to monocytes. These two types of fibrocytes are thought to have a different origin. CCR2^−^ fibrocytes are likely derived from circulating fibrocytes. It is assumed that CCR2^+^ fibrocytes are differentiated from infiltrating CCR2^+^Ly6C^high^ monocytes since: (1) CCL2 was the major chemokine produced in the injured colon in both acute and chronic injury; (2) CD45^+^CD11b^+^CCR2^+^Col I^−^ monocytes/macrophages, as well as fibrocytes in the colonic LP of injured mice, were positive for *Col1a1*; (3) adoptively transferred CD45.2^+^CCR2^+^Ly6C^+^Col I^−^ monocytes differentiated into CD45.2^+^CD11b^+^Col I^+^ fibrocytes in the inflamed colon; and (4) the total monocytes and fibrocytes in the colonic LP after chronic injury significantly decreased, and colon fibrosis was attenuated in CCR2^RFP/RFP^ BM chimeras compared with WT BM chimeras. Shi *et al*. demonstrated that CCR2^+^CD11b^+^Ly6G^int^ monocytic myeloid-derived suppressor cells, which were positive for Kruppel-like factor 4, differentiated into fibrocytes^57^. Wang *et al*. reported that intramuscular CCR2^+^Ly6C^low/−^F4/80^+^ fibrocytes originated from infiltrating monocytes/macrophages, differentiated within injured muscles and played a pathological role in maintaining chronic inflammation and driving progressive fibrosis in a mouse model for Duchenne muscular dystrophy^58^. Their findings are very similar to ours. Although the two types of fibrocytes in our study may play different roles during wound repair and fibrosis, CCR2^−^ fibrocytes might not be associated with the development of colon fibrosis as a substantial number of them remained in CCR2^RFP/RFP^ BM chimeras in which colon fibrosis was significantly reduced.

Recently, BM-derived fibrocytes have been reported to differentiate into fibroblasts and myofibroblasts and participate in tissue wound repair and fibrosis^16,20–22,59^. Suga *et al*. reported that BM-derived fibrocytes could contribute to the myofibroblast population at the wound site in the acute but not in the chronic phase after injury^20^. Uehara *et al*. also described that α-SMA^+^ myofibroblasts appeared during days 7–14 in a murine model of DSS-induced colitis^26^. However, the origin of α-SMA^+^ myofibroblasts were not clarified; the authors suggested that BM transplantation studies using the Y chromosome or green fluorescent protein as a marker of donor cells would be necessary. In our study, a considerable number of EGFP^+^ fibrocytes existed in the injured colon, but there were only a few EGFP^+^ fibroblasts and myofibroblasts during the fibrosis process. Some researchers have demonstrated that the contribution of fibrocytes to myofibroblasts is small in the lung, kidney and muscle fibrosis murine models^10,58,60,61^.

Some authors reported that the number of fibroblasts increased in both the mucosa and submucosa of the colon of mice chronically treated with DSS compared with the untreated control mice^62,63^. Fibrocytes in the colonic LP significantly increased during chronic DSS treatment. However, in our study, there was no change in the number of fibroblasts, most of which were resident cells, in EGFP BM chimeras. Although CCR2 deficiency did not affect the number of fibroblasts in the colonic LP of the chronically treated mice, it reduced the number of fibrocytes and the development of colon fibrosis. Tsukui *et al*. also reported that the number of fibroblasts in the lungs remained constant throughout bleomycin-induced lung fibrosis, and excessive deposition of ECM during lung fibrosis cannot be accounted for by an increase in fibroblast numbers^61^. Furthermore, they supposed that fibrocytes were the subsets most likely to contribute to fibrotic responses in the injured lung because CCR2-deficient mice were protected from fibrosis. Their results and ours suggest that the development of tissue fibrosis is significantly associated with the accumulation of CCR2^+^ cells, especially Ly6C^high^ monocytes and their progenies, fibrocytes.

Although there was no significant difference in the expression level of *Col1a1* and *Tgfb1* in the colon between EGFP BM and CCR2^RFP/RFP^ BM chimeras after three cycles of DSS treatment, the fibrosis area assessed by Sirius red staining was significantly reduced in the CCR2^RFP/RFP^ BM chimeras. These results evoke the possibility that the reduction of Col I deposition may be caused by the stimulation of Col I degradation rather than the inhibition of Col I synthesis.

When we compared the expression level of *Mmp1a*, *Mmp8*, *Mmp13* and *Timp1* within the colon tissues after three cycles of DSS treatment between EGFP BM and CCR2^RFP/RFP^ BM chimeras, only *Timp1* expression was significantly reduced in CCR2^RFP/RFP^ BM chimeras. An increased level of TIMP-1 protein has been reported in inflammatory and fibrotic lesions in Crohn’s disease and a murine model of chronic inflammation-induced intestinal fibrosis^43,44^. Breynaert *et al*. reported that in the chronic colitis model, reduced colonic inflammation, lower tissue collagen levels and less fibrosis were observed in TIMP-1 knockout mice compared with WT mice^64^. Therefore, TIMP-1 may be a promising therapeutic target to prevent the progression of colon fibrosis. Macrophages, neutrophils, eosinophils, fibroblasts and myofibroblasts reportedly produce TIMPs within the colonic LP^34,44^. We found that fibrocytes, CD11b^+^CCR2^+^ cells and fibroblasts, but not CD11b^+^CCR2^−^ cells expressed *Timp1*, and TIMP-1^+^ cells significantly decreased in CCR2^RFP/RFP^ BM chimeras compared with EGFP BM chimeras after chronic DSS treatment. These results show that TIMP-1, which is synthesised by CCR2^+^ cells, such as monocytes, macrophages and fibrocytes, plays a crucial role in the development of colon fibrosis.

Recently, Friedman *et al*. reported that cenicriviroc, a dual antagonist of CCR2 and CCR5, showed a significant benefit in patients with non-alcoholic steatohepatitis with liver fibrosis^65^. Furthermore, angiotensin II type 1 receptor blockers, which are globally used for the treatment of hypertension, were also reported to act as CCR2 antagonists^66^. In the future, these drugs might be useful for the treatment of patients with colon fibrosis.

In conclusion, our results suggest that circulating CCR2^+^Ly6C^high^ monocytes migrate into the inflamed colon via the CCL2/CCR2 axis, differentiate into CCR2^+^Ly6C^−^F4/80^+^ fibrocytes and contribute to the development of colon fibrosis through the production of TIMP-1. Therefore, blocking the migration of CCR2^+^Ly6C^high^ monocytes to the inflamed colon may be a potential new therapeutic option to prevent colon fibrosis in patients with IBD. Although colon fibrosis was attenuated with the reduction of CCR2^+^ monocyte-derived cells, it was not completely inhibited, as seen in Fig. 7B. As multiple intestinal cell types other than CCR2^+^ monocyte-derived cells may contribute to the development of colon fibrosis, further experiments are necessary to clarify the mechanism underlying colon fibrosis and develop more effective and targeted therapeutic strategies for colon fibrosis.

## Materials and Methods

### Mice

WT C57BL/6-Ly5.2 mice were purchased from SLC (Shizuoka, Japan). Breeding pairs of WT C57BL/6J-Ly5.1, CCR2-deficient (CCR2^RFP/RFP^; C57BL/6-Ly5.2 background) and CX3CR1-deficient (CX3CR1^GFP/GFP^; C57BL/6-Ly5.2 background) mice were purchased from Jackson Laboratories (Bar Harber, ME). Breeding pairs of EGFP mice (C57BL/6-Ly5.2 background) were kindly provided by Dr. M. Okabe (Osaka University, Japan)^67^. WT C57BL/6J-Ly5.1, CCR2^RFP/RFP^, CX3CR1^GFP/GFP^ and EGFP mice were bred and maintained at the Institute of Laboratory Animals, Mie University. CCR2^RFP/+^CX3CR1^GFP/+^ mice were obtained by crossing CCR2^RFP/RFP^ and CX3CR1^GFP/GFP^ mice^52^. The experimental protocol was approved by Animal Research Committee, Mie University (approval number: 27-10). All animal studies were conducted in accordance with the institutional guidelines and regulations for animal experiments.

### BM transplantation

First, 10- to 14-week-old male C57BL/6J-Ly5.1 mice were irradiated with a single 10-Gy dose of total irradiation using a 4 × 10^6^ V linear accelerator. Then, 5 × 10^6^ BM-TNCs obtained from 10- to 14-week-old female EGFP, C57BL/6-Ly5.2, or CCR2^RFP/RFP^ mice were injected into irradiated male C57BL/6J-Ly5.1 mice. Recipient mice were fed sterile chow and water without antibiotics. We observed 91.2%–99.3% donor cell (EGFP^+^ or CD45.2^+^ cells) chimerism within the total leukocytes of the PB as assessed by flow cytometry for both donor cell types in mice at 2 months after BM transplantation (data not shown).

### Induction of colitis

We confirmed that colon length did not change between unirradiated and irradiated C57BL/6-Ly5.1 mice at 2 months after BM transplantation (data not shown), in agreement with the report by Followill *et al*., who reported that single doses of less than 20 Gy produced acute crypt cell depletion followed by successful regeneration, repopulation and restoration of the colonic mucosa^68^. Therefore, AOM/DSS treatment was started at 2 months after BM transplantation. To establish the appropriate concentration of DSS in BM chimeras, different amounts (1% and 2%) of DSS (MW 36–50 kDa, MP Biomedicals, Santa Ana, CA) dissolved in drinking water were administered for 7 days, followed by water alone for 2 weeks. Almost all mice died immediately after 2% DSS administration; thus, colitis was induced using 1% DSS. Mice were injected with 10 mg/kg AOM (Wako Pure Chemical Industries, Osaka, Japan) intraperitoneally 1 week before DSS treatment, and DSS treatment was repeated for 3 cycles. Healthy control mice received only water. AOM/DSS-induced colitis was scored as DAI, which was the combined score of weight loss, stool consistency and bleeding^69^. DAI score was monitored three times per week. Non-transplanted CCR2^RFP/+^CX3CR1^GFP/+^ mice were administered 2% DSS because their DAI scores were significantly lower than those of BM chimeras when both groups of mice were treated with 1% DSS.

### Tissue preparation

Mice were euthanised by cervical dislocation after anaesthesia with isoflurane, and their colons were resected and fixed in 4% phosphate-buffered paraformaldehyde for 1 h at room temperature. Some tissue blocks were embedded in paraffin after dehydration in a graded alcohol series. Other tissue blocks were fixed in 4% phosphate-buffered paraformaldehyde for another 5 h at room temperature, embedded in Tissue-Tek OCT medium (Sakura Finetek USA, Torrance, CA), rapidly frozen by plunging into liquid nitrogen and stored at −80 °C. Tissue blocks were cut to 5 μm sections using a microtome or a cryostat.

### Histological analysis

Serial sections were stained with HE, and the histological inflammation score was determined as previously described^69^. Each sample was randomly selected from three perspectives, and average scores were calculated. The sections were stained with Sirius red and Masson’s trichrome to evaluate the presence of fibrosis. Three images per section were captured at 100× magnification using an Olympus BX41 microscope (Olympus, Tokyo, Japan) equipped with a 10×/0.40 numerical aperture objective lens and an Olympus Camedia C-5060 camera. Image J software (NIH, Bethesda, MD) was used for evaluating the percentage of red staining for Sirius red and that of blue staining for Masson’s trichrome in the whole area of each image, with the dyed area indicating the presence of collagen fibres in the tissue. Sections stained with Sirius red were analysed using an Olympus BX50 microscope fitted with polarising filters to quantify type I and type III collagen. Photomicrographs were obtained in 100× magnification in three representative fields for each sample. The counting of collagen bundles was performed using Image J software and the percentages of red staining for type I collagen and of green staining for type III collagen in the whole area of each image were calculated.

### Isolation of PB cells

Blood was collected from anaesthetised mice via cardiac puncture using a heparinised syringe. After red blood cell removal using ammonium-chloride-potassium lysis buffer, white blood cells were pelleted by centrifugation at 500 × g for 10 min at room temperature and resuspended in 400–600 µL of Ca^2+^- and Mg^2+^-free phosphate-buffered saline (PBS^−^) containing 0.1% bovine serum albumin (BSA).

### Isolation of colonic LP cells

Colonic LP cells were collected from colons, as previously described^70^. The colons were resected, and their length, from the ileocecal junction to the rectum, was measured. The colons were opened longitudinally, washed with saline to remove intestinal contents and weighed. After measurements, they were cut into 1.0-cm pieces, which were incubated with Hanks’ balanced salt solution (HBSS) lacking Ca^2+^ and Mg^2+^ and containing 2.5% foetal calf serum (FCS), 1 mM dithiothreitol and 1% penicillin/streptomycin/glutamine, followed by warming in a water bath at 37 °C for 5 min and shaking (200 rpm) at 37 °C for 15 min to remove mucus. Subsequently, epithelial cells were removed through incubation with HBSS containing 2.5% FCS, 1 mM ethylenediaminetetraacetic acid (Invitrogen, Carlsbad, CA) and 1% penicillin/streptomycin/glutamine by warming in a water bath at 37 °C for 5 min and shaking (200 rpm) at 37 °C for 30 min; this procedure was performed twice. The colonic pieces were then digested in HBSS containing 2.5% FCS, 1.5 mg/ml collagenase VIII (Sigma-Aldrich, St. Louis, MO) and 0.1 mg/ml DNase I (Worthington Biochemical, Lakewood, NJ) by shaking (200 rpm) at 37 °C for 30 min. The resultant cell suspensions were sequentially passed through cell strainers (70 μm), resuspended in 40% Percoll (GE Healthcare UK, Little Chalfont, UK) and layered on top of 75% Percoll before centrifugation at 2500 rpm for 20 min at room temperature. Cells residing at the interface of 75% and 40% Percoll layers were collected, washed twice with PBS^−^ and resuspended in 0.1% BSA PBS^−^ for use in further experiments.

### Immunohistochemical analysis

Frozen colon sections were treated with 0.5% Triton X-100 in PBS^−^ for 1 h, and non-specific binding was blocked with 3% BSA in PBS^−^ for 1 h. Frozen colon sections from EGFP BM chimeras were incubated with phycoerythrin (PE)-conjugated rat anti-mouse CD45 (BD Biosciences Pharmingen, San Diego, CA) to detect haematopoietic lineage cells. After treatment with the M.O.M. Immunodetection Kit (Vector Laboratories, Burlingame, CA), the frozen sections were stained with cyanine (Cy) 3-conjugated anti-α-SMA (Sigma-Aldrich). Staining with PE-conjugated isotype control (BioLegend, San Diego, CA) or Cy3-conjugated isotype control (Jackson Laboratories) was performed as a negative control. Frozen colon sections from EGFP BM or CCR2^RFP/RFP^ BM chimeras were stained with anti-TIMP-1 (R&D Systems, Minneapolis, MN), followed by Alexa Fluor 568- or Alexa Fluor 488-conjugated donkey anti-goat IgG (Molecular Probes Invitrogen, Carlsbad, CA). Nuclei were stained with TOPRO3 iodide (Life Technologies, Eugene, OR). Triple-fluorescence immunohistochemical analysis was performed by incubating the frozen sections with anti-procollagen1A1 (Santa Cruz Biotechnology, Dallas, TX), followed by Alexa Fluor 568 conjugated donkey anti-goat IgG and allophycocyanin (APC)-conjugated rat anti-mouse CD45 (eBioscience, Santa Clara, CA). Staining without primary antibodies, followed by Alexa Fluor 568-conjugated donkey anti-goat IgG and APC-conjugated isotype control (BioLegend), was performed as a negative control. All sections were examined using an Olympus IX81 FV1000 laser scanning confocal microscope.

### Flow cytometry analysis

Colonic LP cells were incubated with anti-mouse CD16/CD32 (BioLegend) to block non-specific Fc receptors, followed by a cell surface staining with the corresponding mixture of fluorescently-labelled monoclonal antibodies. Seven-amino actinomycin D (BioLegend) was used to discriminate live and dead cells. The following antibodies conjugated with biotin, PE, peridinin chlorophyll-Cy5.5, PE-Cy7, APC, APC-Cy7, brilliant violet (BV) 421, BV510, BV650, BV711, or BV785 were used for flow cytometry: anti-B220, anti-CD4, anti-IA/IE, anti-Ly6G, anti-Siglec F (all from BD Biosciences), anti-CD3e, anti-CD8a, anti-CD11b, anti-CD11c, anti-CD45, anti-CD45.2, anti-CXCR4, anti-F4/80, anti-TER119 (all from BioLegend), anti-Ly6C, anti-NK-1.1, anti-Siglec F (all from Miltenyi Biotec, Auburn, CA) and anti-CCR2 (R&D Systems). Following extracellular staining, the cells were fixed and permeabilised with Cytofix/Cytoperm (BD Biosciences) and sequentially incubated with rabbit anti-collagen type I (Rockland, Limerick, PA), followed by Alexa Fluor 647-conjugated goat anti-rabbit IgG (Invitrogen). Data were acquired on LSRFortessa and processed using FlowJo software (Tree Star, Ashland, OR) with appropriate isotype controls to determine gating. Cell sorting was performed using FACSAria II (BD Biosciences).

### RNA isolation

After cutting the colon in longitudinally, 0.5 cm of the rectum was collected from the anal side, placed in RNAlater (Invitrogen) and stored at 4 °C. RNA was extracted using the RNeasy Mini Kit (Qiagen, Valencia, CA) according to the manufacturer’s protocol.

RNA was isolated from colonic LP cells following intracellular antibody staining and fluorescence-activated cell sorting according to a previously reported method^71^. After sorting, cells were pelleted by centrifugation at 2000 × g for 15 min at 4 °C. Total RNA was isolated from the pellet using the RecoverAll Total Nucleic Acid Isolation Kit (Ambion, Austin, TX), beginning with the protease digestion step of the manufacturer’s recommended protocol. The isolation procedure was modified as follows: cells were incubated in digestion buffer at 50 °C for 3 h, cell lysates were frozen at −80 °C overnight and RNA was isolated according to the manufacturer’s instructions.

### Quantitative RT-PCR

Complementary DNA was generated from total RNA using the SuperScript III First-Strand Synthesis System for RT-PCR (Invitrogen) according to the manufacturer’s protocol. The synthesised complementary DNA was quantified by RT-PCR using TaqMan Gene Expression Master Mix (Applied Biosystems, Carlsbad, CA) and TaqMan probes. Reactions were run on a StepOnePlus Real-Time PCR System Upgrade (Applied Biosystems) with default settings. The following primers were used: *Ccl2* (Mm00441242_m1), *Cxcl12* (Mm00445553_m1), *Cx3cl1* (Mm00436454_m1), *Col1a1* (Mm00801666_g1), *Tgfb1* (Mm01178820_m1), *Timp1* (Mm01341361_m1), *Mmp1a* (Mm00473485_m1), *Mmp8* (Mm00439509_m1), *Mmp13* (Mm00439491_m1) and *Gapdh* (Mm99999915_g1). The relative expression of each gene was normalised to that of the housekeeping gene *Gapdh*.

### Adoptive transfer of BM monocytes

In the adoptive transfer experiment, colitis was induced using 1% DSS in non-transplanted C57BL/6J-Ly5.1 mice. Monocytes (3–4 × 10^6^ cells per mouse), which were isolated from BM-TNCs of WT C57BL/6J-Ly5.2 or CCR2^RFP/RFP^ mice using the EasySep Mouse Monocyte Isolation Kit (STEMCELL Technologies, Vancouver, Canada), were intravenously administered into DSS-treated mice at 8 and 13 days after initiation of DSS treatment. Recipient mice were sacrificed at day 16 after colitis induction.

### Statistics

Data are expressed as the mean and standard deviation. Two experimental groups were compared using Mann–Whitney U test. For the comparison of more than two groups, Kruskal–Wallis test followed by Dunn’s multiple comparison test was performed. Analyses were performed using Prism software (GraphPad Software, La Jolla, CA). A *p*-value of <0.05 was considered statistically significant.
